# Multiple objects interacting with a solidification front

**DOI:** 10.1038/s41598-021-82713-3

**Published:** 2021-02-10

**Authors:** Sidhanth Tyagi, Cécile Monteux, Sylvain Deville

**Affiliations:** 1 Laboratoire de Synthèse et Fonctionnalisation des Céramiques, UMR 3080 CNRS/Saint-Gobain CREE, Saint-Gobain Research Provence, Cavaillon, France; 2 Sciences et Ingénierie de la Matiére Molle, ESPCI Paris, PSL Research University, CNRS, Sorbonne Universités, UPMC Univ Paris 06, Paris, France; 3 Global Station for Soft Matter, Global Institution for Collaborative Research and Education, Hokkaido University, Sapporo, Japan; 4 Université de Lyon, Université Claude Bernard Lyon 1, CNRS, Institut Lumière Matière, 69622 Villeurbanne, France

**Keywords:** Physical chemistry, Imaging techniques, Materials science, Soft materials

## Abstract

The interaction of objects suspended in a liquid melt with an advancing solidification front is of special interest in nature and engineering sciences. The front can either engulf the object into the growing crystal or repel it. Therefore, the object-front confrontation can have a strong influence on the microstructure and mechanical or functional properties of the solidified material. The past theoretical models and experimental studies have mostly investigated the interaction of isolated, spherical, and hard objects in pure melts. However, the outcome of object-front interactions in complex (more realistic) systems, where multiple objects and solutes are present, is still poorly understood. Here we show the interaction of multiple oil droplets with an ice-water front in the absence and presence of solute effects using in situ cryo-confocal microscopy. We report on how the object size, number of objects, and bulk solute concentration influence the the object-front interaction and the front morphology, as well as the subsequent object spatial distribution. We suggest that the volume fraction of objects suspended in a liquid melt in conjunction with the amount of bulk solute concentration are two important parameters to be incorporated in the development of object-front interaction models.

## Introduction

The interaction of soft (bubbles, droplets, cells) or hard (rigid particles) objects with a moving solidification front is a ubiquitous phenomenon with diverse natural and technical occurrences. In nature, the formation and growth of sea ice, frost heave in cold regions^1,2^, while the technological incidences include cryobiology^3,4^, food engineering^5^, metallurgy^6^, growth of single crystals^7,8^, and ice-templating^9^. At the core of these physical processes are objects (particle, droplet, or bubble) encountering an approaching solid–liquid front. A major goal in understanding these systems is to elucidate the outcome of this interaction and predict not only the object behaviour (engulfment or rejection) but also the spatial distribution of objects after solidification. The solidification microstructures obtained owing to this object-front confrontation are vital as they govern the mechanical and functional properties of the solidified material. In metal-matrix-composites, a homogeneous distribution of reinforcing particles improves the structural properties, while a particle segregation along the grain-boundaries is detrimental to their performance^10^. In contrast, an in situ evolution and trapping of gas bubbles in metals and single crystals can lead to their catastrophic failure and hence, a total rejection of bubbles is desired^11^.

The first systematic measurements of particle interaction with an advancing ice-water front were conducted by Corte^12^. The objective of the experiments was to study the influence of an increasing growth rate ($V_{sl}$) on particle capture by the growing solid. Studies performed subsequently on the interaction of a single isolated object with a solidification front deduced a critical velocity ($V_c$), the growth rate below which an object is pushed ($V_{sl}<V_c$), while the object is engulfed by the growing solid above it ($V_{sl}>V_c$)^13,14^. The previous studies report that the precise value of $V_c$ depends on several factors, comprising of the particle radius *R*, the temperature gradient *G*, and the physical characteristics of the particle-melt-crystal system^15–17^. In general, $V_c\propto R^{-\alpha }$ where $\alpha$ varies ($1\le \alpha \le \frac{3}{2}$) owing to an interplay between the viscous drag forces ($F_{\eta }$), which promote capture, and the thermomolecular forces ($F_{\sigma }$), which favor repulsion of the object through a premelted film^16–19^.

A plethora of work has been performed to determine theoretically the magnitude of these opposing forces, which have a major dependence on the shape of the solidification front^10,20,21^. The studies only differ in the theoretical approximations used (e.g. boundary conditions) and mainly deal with an isolated, smooth, spherical, and rigid particle of radius *R* moving ahead of a planar front in steady-state (i.e. $F_{\eta }=F_{\sigma }$) directional solidification^15^. The in situ experimental correlations of these analytical and numerical models are, however, still scarce owing to the numerous complexities associated with solidification, such as multiplicity of space- and timescales^4,11,15,22–25^. Furthermore, the idealistic assumptions of the theoretical models such as the non-retarded van der Waals forces, absence of solute segregation, and a steady-state planar front interaction are difficult to achieve practically^26^. The advent of accurate finite element models also clearly highlights the disparities in approximations to solidification front shapes and the accuracy of the simulated particle behaviours^27^.

**Figure 1:**
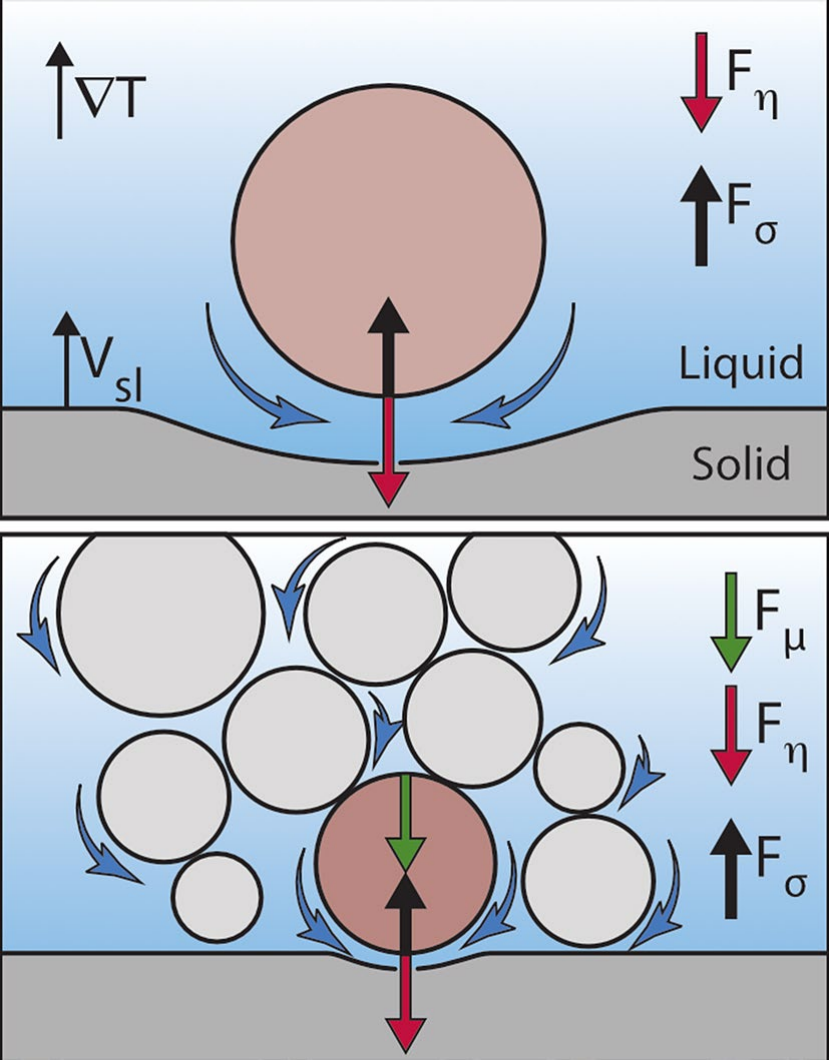
Force equilibrium to predict engulfment or rejection of objects in the presence of multiple particles. The particle–particle interactions during solidification can result in the formation of a compact layer, which acts as a porous medium. The subsequent resistance offered to the flow of fluid (blue arrows) towards the growing solid results in an additional frictional force $F_{\mu }$, which favors object engulfment. The modified force equilibrium is hence given as $F_{\eta }+F_{\mu }=F_{\sigma }$, where $F_{\eta }$ is an attractive viscous drag force and $F_{\sigma }$ is a repulsive interfacial force. $\nabla T = G$ is the applied temperature gradient and $V_{sl}$ is the imposed growth rate. $^{\copyright }$ (2020) S. Tyagi et al. (10.6084/m9.figshare.13176221) CC BY 4.0 license https://creativecommons.org/licenses/by/4.0/.

The existing theories and experimental evidences have been primarily derived for isolated objects, whereas most real-life applications involve multiple polydisperse particles. This reinforces the importance of investigating the role of particle size distribution, particle–particle interactions and hence, the formation of agglomerates. Progress in studying the spatial distribution, when thousands of particles interact with a solid–liquid front, has been brought about by modern advanced in situ imaging techniques, such as X-ray imaging^28^, confocal microscopy^29^, or the development of numerical modelling techniques, such as molecular dynamics^30,31^. Recently, Saint-Michel et al.^32^ proposed a mechanical model on the effects of multiple particle interactions on particle trapping by solidification fronts as depicted in Fig. 1. Their study highlighted the major differences in force equilibrium when considering a multi-particle approach. Their experimental results revealed that the presence of multiple particles can lead to an accumulated particle layer ahead of the solidification front at small growth rates ($V_{sl}<V_c$). This particle layer further acts as a porous medium and offers resistance to the fluid flow resulting in an additional frictional force ($F_{\mu }$), which facilitates particle engulfment. Hence, the modified force equilibrium for multiple-particles can be given as $F_{\eta }+F_{\mu }=F_{\sigma }$. Although, these studies help us to bridge the gap between the single particle models and real-life multi-particle systems, we now need to understand how the presence of multiple particles at the front can locally affect the solidification microstructure.

The presence of solutes, desired or as impurities, impacts the solidification front shape and induces long-range effects (at typical length scales of $\approx D/V_{sl}$, where *D* is the solute diffusion coefficient), which override the short-range thermomolecular forces ($\le 10$ nm). The existing models, incorporating the role of solute, study their influence on the solid–liquid front shape deformation but not on the nature and equilibrium of the repulsive forces^19,21,33–35^. In general, the solubility of a solute is less in the solid phase than in the liquid phase. As solidification progresses, the solute is rejected by the growing crystal, thereby corresponding to an increasing concentration of solute in the remaining liquid^36^. In the presence of foreign objects, the segregation of solutes at the front is enhanced further by the approaching objects obstructing their diffusion field^21^. This local solute enrichment influences the object environment, (due to local undercooling), and is of particular importance in understanding several mechanisms that can occur at the solid–liquid front. The rejection of additives during the freezing of biological cells can inflict membrane rupture (or cryoinjury) owing to the high osmotic stress gradient from the local solute concentration^3,4^.

The objective of our study is to investigate experimentally the solidification dynamics and spatial distribution of objects in multiple particle regimes. We compare our experimental observations with previous theoretical models to comprehend the critical correlations and disparities in the presence of multiple particles. We perform directional solidification experiments with independently regulated solidification velocity ($V_{sl}$) and a linear temperature gradient (*G*). Both the solidification velocity and the temperature gradient are constant during the entire experiment. The use of freezing emulsions as model system for solidification has been promising^37^ and we develop further on this approach using in situ cryo-confocal microscopy. We utilise mono-disperse, bimodal emulsions, and subsequently progress towards realistic poly-disperse systems. We demonstrate several important aspects of multiple particle interactions and its consequences on particle redistribution in the solidified microstructure. We increase the complexity by adding solute to the liquid phase, and examine the effects of increasing poly-dispersity in conjunction with the overriding solute effects. Finally, we illustrate how the systematic approach facilitates to decouple the process parameters impacting the behaviour of an object, while it interacts with a solid–liquid front.

## Experimental methods

### Materials

We purchased propyl benzoate, TWEEN 80, Difluoro2-[1-(3,5-dimethyl-2H-pyrrol-2-ylidene-N)ethyl]-3,5-dimethyl-1H-pyrrolato-Nboron (BODIPY), and Sulforhodamine B (SRhB) from Sigma-Aldrich. We utilised 0.45 μm Nylon membrane filters (VWR International) for filtering traces of impurities in the deionized water used for the aqueous phase. We chose propyl benzoate for the oil phase as it has a low melting temperature ($T_m= -51.6$ °C), low solubility in water ($0.035\,{\rm g}/100\,{\rm g}$), and similar density to water ($\rho _{oil}=1.023\,{\text{g}}\cdot {\rm cm}^{-3}$).

### Sample preparation

We prepared oil-in-water emulsions using a microfluidic setup (microfluidic starter kit, Micronit Microfluidics, Netherlands) with pressure controlled flow pumps (Fluigent LineUP Flow EZ). We used uncoated focused flow droplet generator chips (FF$\_$DROP, Micronit) with nozzle diameter of 10 μm and 50 μm. These microfluidic chips facilitated the preparation of highly monodisperse droplets with radii of 8 μm ($R_1$) and 28 μm ($R_2$), respectively. We ensured a uniform flow rate of oil and aqueous phases using Fluigent Flow Unit S ($0-7\,\upmu {\rm {L\cdot min^{-1}}}$) combined with in-line impurity filters (PEEK 2 μm,VWR International). The oil phase consisted of propyl benzoate with $10^{-4}\,M$ BODIPY to obtain clear imaging of dispersed droplets at $1\%$ laser power. For the aqueous phase, we used $10^{-5}\,{\text{M}}$ SRhB solution, as self-quenching was reported at concentrations above $2\times 10^{-4}\,{\text{M}}$^38^. We added TWEEN 80 ($HLB=15$^39^), a non-ionic surfactant, to the aqueous phase for stabilising the oil-in-water emulsions. TWEEN 80 (Critical Micellar Concentration = $13-15\,{\rm mg}\cdot {\rm l}^{-1}$^40^), acted as a model solute as it depresses the freezing point of solutions alike (colligative property). The bimodal emulsions were synthesized by independently mixing the monodisperse emulsions ($R_1 + R_2$). The polydisperse emulsion was obtained through hand-shaking the aqueous suspension in a $1.5 \,{\rm ml}$ eppendorf vial with $2\,{\text{vol}}.\%$ oil phase. The prepared emulsions were filled through capillarity and solidified in a rectangular Hele-Shaw cell (h=100 μm and V=$100\,\upmu {\rm l}$). The cell was fabricated using two glass slides (Menzel, $24\times 60\,{\rm mm}$, thickness $0.13-0.16\,{\rm mm}$), and sealed with nail-polish at one end to prevent evaporation.

### Imaging & analysis

We used Leica TCS SP8 confocal laser scanning microscope (Leica Microsystemes SAS, Germany), equipped with ${488\,{\rm nm}}$ (blue) and ${552\,{\rm nm}}$ (green) lasers, for image acquisition. In 2D, we utilised the microscope at a scanning speed of $600\,{\rm Hz}$, with $1024\times 1024\,pixels$ for imaging $775\times 775$ μm, resulting in $1.7\,{\rm s}$ per frame. In 3D, we used a fast resonant mode with $512\times 512\,pixels$ for a scanning rate of $0.047\,{\rm s}$ per frame. We used two photodetectors (PMT) to simultaneously image three phases :

1. BODIPY (${\lambda _{ex}}$ ${493\,{\rm nm}}$ ; ${\lambda _{em}}$ ${504\,{\rm nm}}$), fluorophore incorporated into the oil droplets.
2. SRhB (${\lambda _{ex}}$ ${565\,{\rm nm}}$ ; ${\lambda _{em}}$ ${586\,{\rm nm}}$), fluorophore dissolved in water, to image the aqueous phase and the cells boundaries in ice.
3. Ice, does not fluoresce, as it has very low solubility for solutes^41^ and hence, appears black.

The emission spectra of the excited fluorophores was captured using a non-immersive objective (Leica HCX PL APO CS $20\times$). The objective working distance of 590 μm along with an insulating foam cladding facilitates the minimization of thermal perturbations on the freezing substrate. We used Fiji^42^ for image thresholding in conjunction with Python^43^ for image and data analysis.

### Freezing stage

We conducted unidirectional solidification experiments, translating the sample cell along a constant linear temperature gradient (*G*), using the cryo-confocal stage described in detail previously^29^. We imposed the temperature with two Peltier modules, and controlled it with high precision ($< 0.01$ °C) using TEC-1122 Dual Thermo Electric Cooling Temperature Controller from Meerstetter Engineering, Switzerland. The Peltier elements were separated by a distance ($2\,{\rm mm}$) to establish a linear temperature gradient along $\vec {x}$. We utilised the VT-80 translation stage (Micos Pollux Drive PI, USA) to impose the rate at the which the sample cell is pulled ($V_{sl}$). The rate of translation was verified to be in agreement with the measured solidification velocity using posterior image analysis ($error < 1\,\%$). Thus, we can control independently the solidification velocity ($V_{sl}$) and the thermal gradient (*G*) in our system. We carried out the experiments in the velocity range of $1\le \,V_{sl}\,\le \,10\,\upmu {\text{m}}\cdot {\rm s}^{-1}$, with sufficient waiting time to establish thermal equilibrium along the sample depth ($\vec {z}$). The in situ observation of object interaction with the solid–liquid front was captured using a confocal microscope placed vertically over the gap ($2\,{\rm mm}$) between the two Peltier modules. Hence, the solidification front tends to appear immobile in the frame of observation, however, in the sample frame, it is the ice solidifying (along $\vec {x}$) at the velocity imposed by the pulling rate of the motor.

## Results

### Front morphology

**Figure 2:**
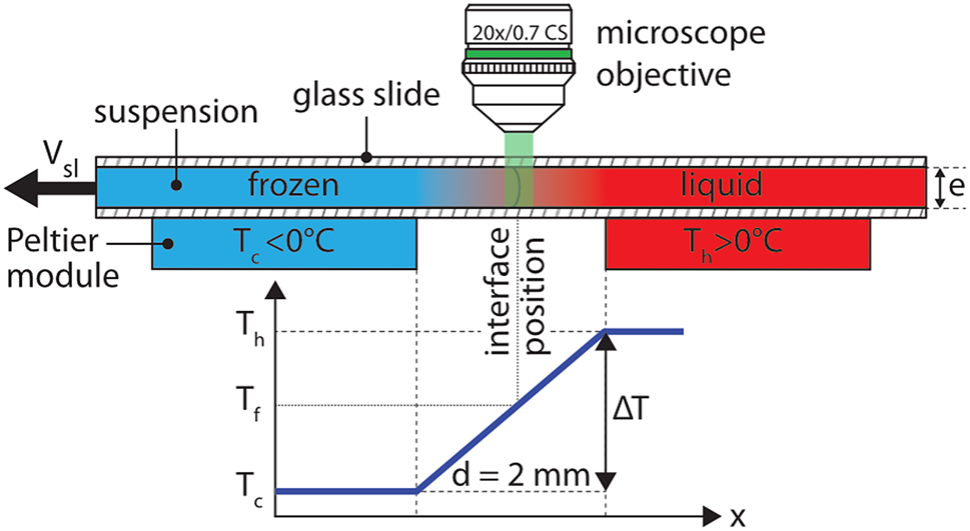
Experimental setup for in situ solidification experiments. A thin Hele-Shaw cell containing the droplets in suspension is pulled at a constant velocity ($V_{sl}$) through a constant temperature gradient (*G*) established by Peltier elements. In steady state, the solidification front is thus at a constant position under the microscope objective.$^{\copyright }$ (2020) S. Tyagi et al. (10.6084/m9.figshare.12046560) CC BY 4.0 license https://creativecommons.org/licenses/by/4.0/.

**Figure 3:**
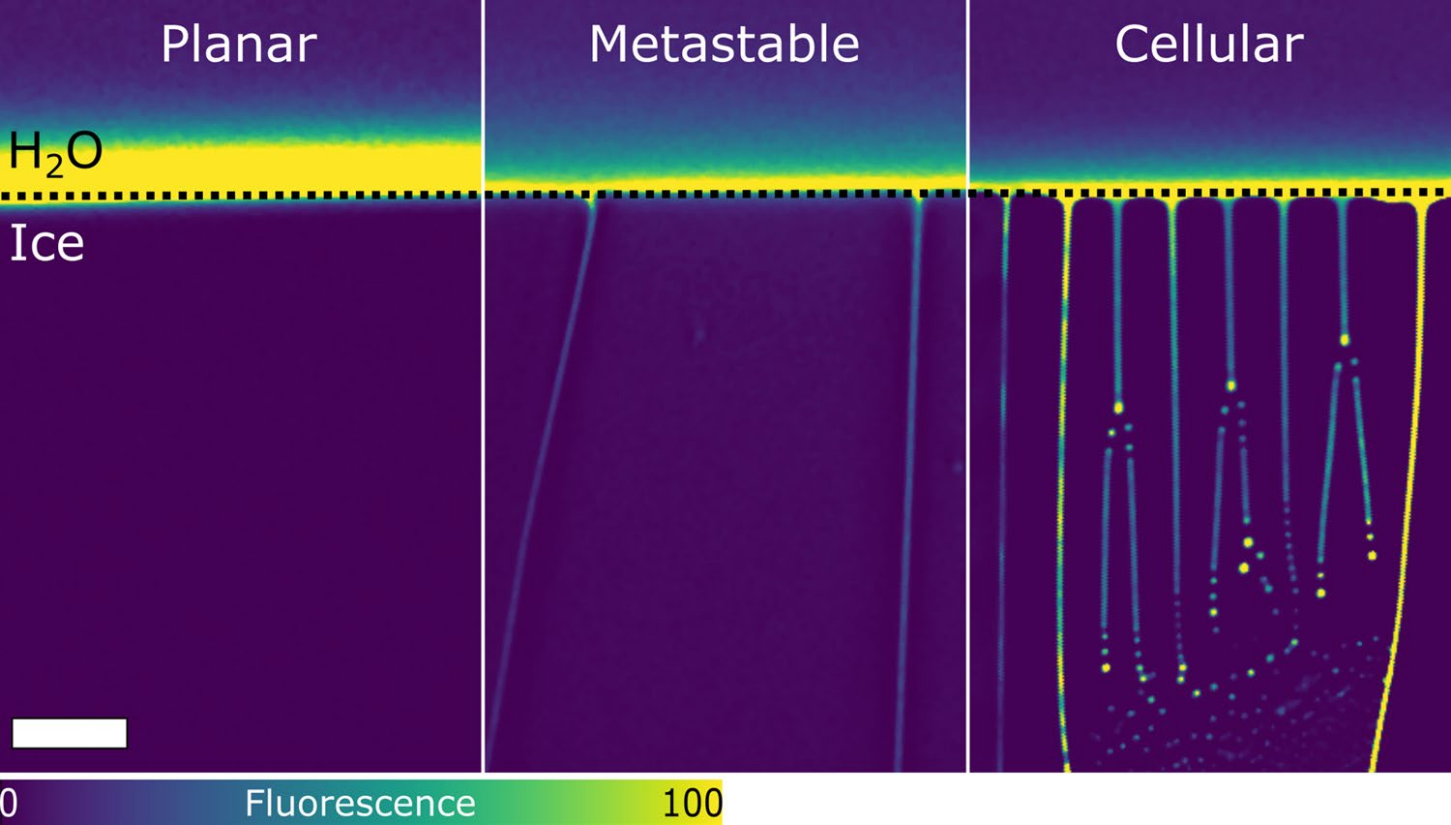
Typical confocal images of directional solidification depicting different front morphologies; planar, metastable, and cellular. Confocal image enables us to distinguish the dark solid phase (ice) from the bright aqueous phase (water). Water is in colormap viridis (fluorescence bar) while ice is in black. Scale bar = $100\,\upmu {\rm m}$. $^{\copyright }$ (2020) S. Tyagi et al. (10.6084/m9.figshare.13176221) CC BY 4.0 license https://creativecommons.org/licenses/by/4.0/.

We study the directional solidification of oil-in-water emulsions, translated at a velocity $V_{sl}$ along a linear temperature gradient *G*, using a confocal microscope, as shown in Fig. 2. A typical confocal image, as shown in Fig. 3, enables us to distinguish the dark solid phase (ice) from the bright aqueous phase (water). Ice, like most solids, has extremely low solubility for dissolved solutes and rejects them in the remaining liquid as solidification progresses^41^. The coexisting solid–liquid phases are delimited by the solidification front, corresponding macroscopically to an isotherm at bulk melting temperature $T_m$.

In directional solidification, the advancing solid–liquid front can have predominantly three morphologies; planar, cellular, and dendritic (Fig. 3). The stability and morphology of a front are essentially controlled by the growth rate, the underlying temperature gradient, and the solute concentration in the melt. The major advantage of our system is that we can independently regulate and accurately maintain a constant solidification velocity ($V_{sl}$) as well as temperature gradient (*G*) over the experimental time-scales. This ensures a steady-state solidification front under a constant cooling rate with the absence of thermal destabilisation.

**Figure 4:**
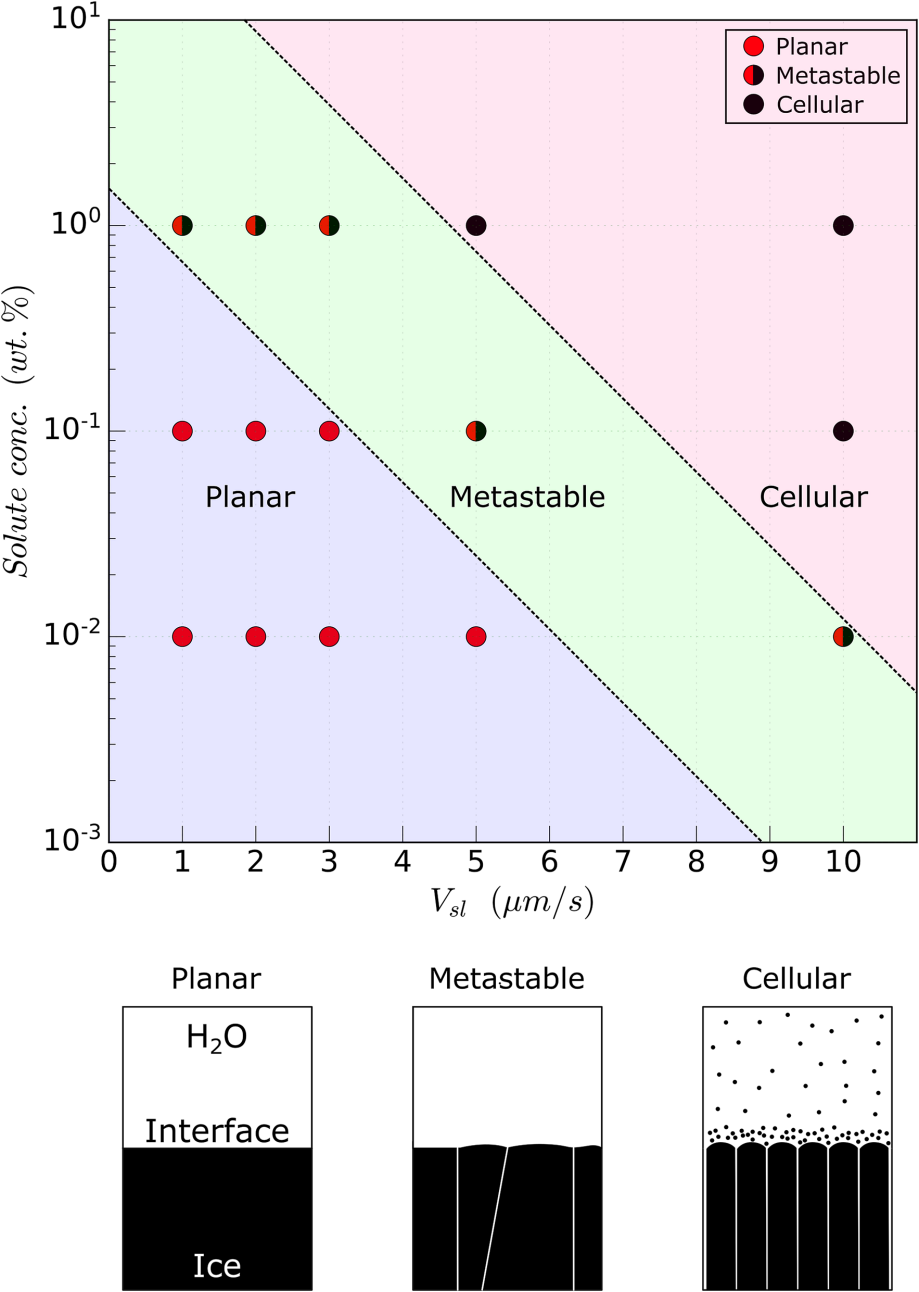
Front morphologies obtained during the directional solidification of an ice-water system in the solute concentration versus growth rate ($V_{sl}$) plane, at a constant thermal gradient of $10^4\,{\rm K}\,{\rm m}^{-1}$. A steady-state planar front gets destabilised partially, with an increasing growth rate and solute concentration, to a metastable front exhibiting a few cells boundaries, and eventually forms a cellular front for the higher solute concentrations ($\ge 10^{-1}$ wt.%). Broken lines are drawn to differentiate the three zones distinctly and do not imply an extrapolation of the experimental data.$^{\copyright }$ (2020) S. Tyagi et al. (10.6084/m9.figshare.13176221) CC BY 4.0 license https://creativecommons.org/licenses/by/4.0/.

To establish the domains of the different front morphologies, we performed experiments using a constant thermal gradient *G* of $10^{4}\,{\rm K}\cdot {\rm m}^{-1}$, as shown in Fig. 4. We observe three distinct front morphologies (Figs. 3, 4), in the absence of suspended objects, while solidifying the aqueous phase in a velocity range of $1\le V_{sl}\le 10\,\upmu {\rm m}\cdot {\rm s}^{-1}$ with varying concentrations of solute. In Fig. 4, the steady-state planar front is thermodynamically favorable at low growth velocities ($V_{sl}\le 5\,\upmu {\rm m}\cdot {\rm s}^{-1}$) for small solute concentrations ($\le 10^{-1}\,$wt.%). As we increase the front velocity or the solute concentration, the steady state planar front develops local growth instabilities with a few cells boundaries, which remain stable during experimental time scales $\approx 40-60\,{\rm min}$. At the higher velocities and solute concentrations, the front eventually destabilises into a cellular front morphology. The uniformly spaced lamellae are generated owing to the propagation of a Mullins-Sekerka instability during the solid–liquid phase transformation^44^.

The solute rejected by the growing ice front builds up a steep concentration gradient with the bulk solution, over a length scale ($\approx D/V_{sl}$)^21,23^ . Since growth from solutions depends on the concentration gradient of solutes, the liquidus temperature of the melt in the vicinity of the front differs from the liquidus temperature of the bulk liquid far from the front. As the solute segregates, the liquidus temperature further decreases. This change in liquid composition alters its transformation temperature, referred to as constitutional undercooling^36^. This constitutional undercooling is a necessary criterion for the destabilization of a planar front into a metastable or cellular front^44^. Theoretically, for steady-state planar front growth the solute concentration gradient in the melt scales as $\nabla C \propto e^{- V_{sl} x / D}$, where *x* is the distance from the solidification front^36^. Therefore, an increasing growth rate leads to a strong increase of the solute concentration gradient. This explains why the critical bulk solute concentration for the destabilization of a planar front decreases with an increasing growth rate, as reported in Fig. 4.

The spatial distribution of the objects in the solidified material is determined by the advancing front morphology obtained at the given experimental conditions^23,45^. In ice-templating, a cellular front is used to segregate the dispersed colloids between the arms of the growing ice crystals^9^. In contrast, the growth of single crystals demands a planar front to ensure a homogeneous structure^7^. We thus feel it is important to investigate the systematic impact of object size, solute concentration, and inter-object interactions on the solidified microstructure with planar and meta-stable fronts. To do so, we modify only the bulk solute concentration, while keeping a constant growth rate of $1\,\upmu {\rm m}\cdot {\rm s}^{-1}$ and a constant temperature gradient of $10^4\,{\rm K}\cdot {\rm m}^{-1}$ during our experiments.

### Interaction of the front with monodisperse objects

**Figure 5:**
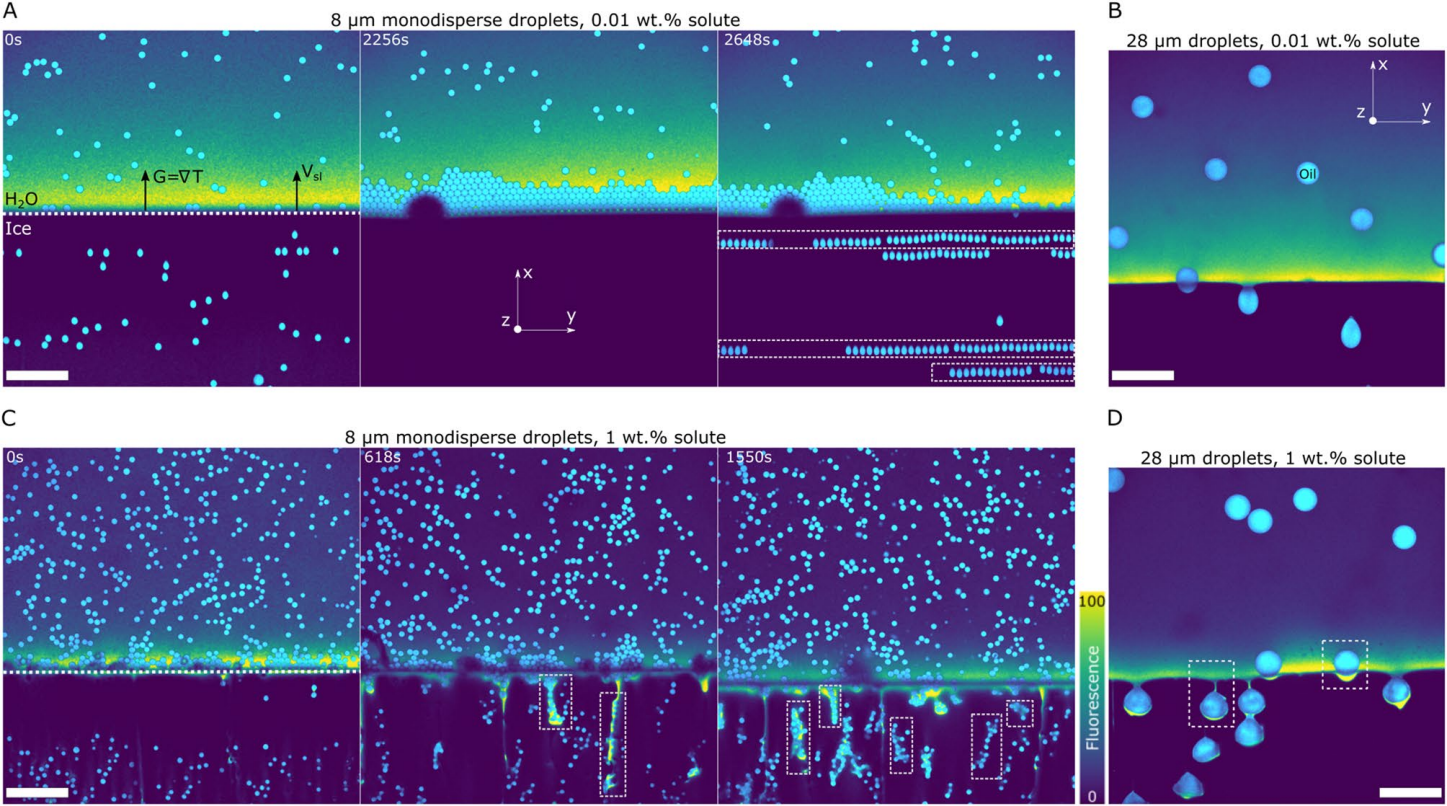
Interaction of monodisperse oil droplets with an advancing ice-water front. Solidification induced features and spatial distribution of oil droplets depicting (**A**) Horizontal mono-layers and stable planar front with $8\,\upmu {\rm m}$ ($R_1$) at $0.01\,$wt.% solute. The semi-circular dark shape is a bubble that happened to be present in this particular experiment. As it does not fluoresce, it appears dark in the experiment. The bubble in this case has no impact on the stability of the front. (**B**) No clusters and stable planar front with $28\,\upmu {\rm m}$ ($R_2$) at $0.01\,$wt.% solute, (**C**) Vertically aligned clusters and local front destabilisation with $8\,\upmu {\rm m}$ ($R_1$) at $1\,$wt.% solute, and (**D**) Rejected solute segregation and local front destabilisation from solute premelted films with $28\,\upmu {\rm m}$ ($R_2$) at $1\,$wt.% solute. Oil is in cyan, water in colormap viridis (fluorescence bar) while ice is in black. Scale bar = $100\,\upmu {\rm m}$. $^{\copyright }$ (2020) S. Tyagi et al. (10.6084/m9.figshare.13176221) CC BY 4.0 license https://creativecommons.org/licenses/by/4.0/.

We first examine the interaction of monodisperse oil-in-water emulsions with the solid–liquid front at a growth rate of $V_{sl}=1\,\upmu {\rm m.s}^{-1}$ and a temperature gradient of $G=10^4\,{\rm K}\cdot {\rm m}^{-1}$, as depicted in Fig. 5. The corresponding droplet size and bulk solute concentration used for the experiments are given in table 1.

**Table 1:** Droplet radius (*R*) and bulk solute concentration (wt.%) in the aqueous phase for the monodisperse oil-in-water emulsions.

Figure 5	*R*	Solute conc.
	$(\upmu {\rm m})$	($wt.\%$)
A	8	0.01
B	28	0.01
C	8	1.00
D	28	1.00

In Fig. 5A, we observe that the $8\,\upmu {\rm m}$ ($R_1$) oil droplets with $0.01\,$wt.% solute concentration are continuously repelled by the ice growing from the bulk suspension, forcing them to build-up as a compacted layer. In our experiment, the height of the close-packed layer increases until $\approx 80\,\upmu {\rm m}$ and subsequently, the droplets get engulfed by the growing solid. Interestingly, the droplets are engulfed as single layers and fabricate a texture parallel to the solid–liquid front. The droplets, albeit closely packed in the liquid ahead of the front, get separated as they are captured by the growing ice crystal. We believe the lateral gap ($\vec {y}$) between the engulfed droplets in ice is created owing to the elongation induced during their capture. The increase in diameter parallel to the temperature gradient ($\vec {x}$) is compensated by the decreasing width in the lateral direction ($\vec {y}$) and hence, the close-packed droplets get separated in the frozen solid. In contrast to the smaller $R_1$ droplets (Fig. 5A), the engulfment of the larger $R_2$ ($28\,\upmu {\rm m}$) droplets is instantaneous and we observe no subsequent compact layer formation (Fig. 5B). The elongation induced owing to the droplet-front interaction is visually prominent during the solidification of the $R_2$ droplets. The deformation of droplets during solidification will be addressed in a separate communication and is not in the scope of the present study. The steady-state planar front remains stable and does not destabilise neither from the formation of the close packed layer nor during the capture of the different size objects (Fig. 5A,B).

The presence of a similar particle agglomerated layer has been reported previously^32,46,47^, where its steady-state thickness (*h*) depends primarily on the solidification velocity ($h\propto V_{sl}^{-1})$. It was shown that the complex scenario of multiple particles can be partly understood by building on the single-particle models. At $0.01\,$wt.% concentration, the solute does not play a dominating role and hence, we shall compare this system to the previous theoretical models derived for single isolated objects. We consider the Rempel-Worster (RW) model^16^, which considers the balance between repulsive van der Waals forces and attractive viscous forces, to estimate the critical particle size ($R_c$), at a given solidification velocity $V_{sl}$, below which particles are rejected by the front and above which particles are captured by an advancing front. The critical particle size $R_c$, can be given as

1 $$\begin{aligned} R_c = \left( \frac{\sigma _{sl} A_{ow}^2}{6^5 \pi ^2 \eta ^3 V_{sl}^3 } \right) ^{1/4} \end{aligned}$$

where $\sigma _{sl}$ is the ice-water interfacial tension, $A_{ow}$ is the oil-water Hamaker constant, and $\eta$ is the dynamical viscosity. We obtain a $R_c$ of $9\,\upmu {\rm m}$ with the typical parameter values (see Table 2) for an object in the ice-water system. Hence, using the RW model we can account for the initial repulsion of the $8\,\upmu {\rm m}$ droplets ($R_1<R_c$) and an instantaneous engulfment of the $28\,\upmu {\rm m}$ ($R_2>R_c$) droplets.

Isolated droplets are rejected at the ice-water front when their size is smaller than the critical size ($R_1<R_c$). In concentrated systems, the droplets being pushed get accumulated ahead of the front and eventually interact with the other droplets, thereby forming a dense object layer (see Fig. 1). Recently, Saint-Michel et al.^32^ have derived a modified force equilibrium for multi-particle systems, and suggested that an additional frictional force, generated by the friction of the fluid flowing through the compact particle layer, toward the ice-water front, favors engulfment above a critical thickness of the packed particle layer. The existence of this critical layer thickness induces periodic repulsion-engulfment transitions as we observe in our study. Thus, we suggest that this additional frictional force, which grows with the height of the compact layer, results in the periodic engulfment of droplets for a critical layer thickness of $\approx 80\,\upmu {\rm m}$.

**Table 2:** Typical solidification parameters for an object in front of an ice-water solidification front. $\sigma _{sl}$ adapted from Rempel et al.^16^.

Parameters	Nominal value
G $(K\cdot m^{-1})$	1.0e4
T$_m$ (*K*)	273.15
$\eta$ $(Pa\cdot s^{-1})$	1.8e−3
$\sigma _{sl}$ $(J\cdot m^{-2})$	3.0e−2
$V_{sl}$ $(m\cdot s^{-1})$	1.0e−6
$A_{ow}$ (*J*)	1.0e−20

We will now discuss the droplet-front interactions with $1\,$wt.% solute in solution. The $8\,\upmu {\rm m}$ ($R_1$) droplets in the presence of $1\,$wt.% solute are pushed by the growing ice and subsequently organise into close-packed clusters (Fig. 5C). The droplet clusters formed are rather non-uniform in their distribution and are heterogeneously scattered over the front in the lateral direction ($\vec {y}$). The droplet clusters are eventually engulfed and incorporated in the growing crystal, while getting textured perpendicular ($\vec {x}$) to the solid–liquid front. This spatial distribution is hence opposite to the parallel mono-layers (along $\vec {y}$) obtained in the presence of $0.01\,$wt.% solute concentration (Fig. 5A). In contrast, the $28\,\upmu {\rm m}$ ($R_2$) droplets get engulfed without repulsion (Fig. 5D), and represent similar behaviour as the corresponding large droplets at $0.01\,$wt.% solute concentration (Fig. 5B). We thus observe a distinct variation in the droplet spatial distribution only in the presence of small $R_1$ droplets.

We believe the formation of these heterogeneous clusters is owing to the local destabilization of the ice front and therefore, instigated by the rejected solute segregation. The presence of multiple droplets further enhances the local solute concentration by obstructing the solute diffusion field in the bulk liquid^13,21,33,34^. Highly fluorescent zones in the vicinity of the droplets can be clearly seen at the front (Fig. 5C,D). These zones are formed owing to the segregation of the fluorescent dye, which is rejected by the growing ice. We expect that the solute, although not fluorescent yet having a similar diffusion coefficient as the dye, segregates in an identical manner at the front. The solute invokes severe constitutional undercooling in the premelted films behind the droplets, thereby inducing a concave curvature of the front^48^.

The extensive concave cusping eventually transforms into a pocket with the droplet build-up and results in subsequent entrapment of droplets along the crystal growth direction ($\vec {x}$). Such solute enriched pockets have also been reported by Chang et al.^49^ during the directional freezing of biological cells. The premelted films are highly sensitive to the concentration of solute and surround the droplets engulfed by the ice, while the interface heals and rebounds to the horizontal isotherm ($T_m$). The presence of solute premelted films with thickness of the order $\approx 5\,\upmu {\rm m}$ leads us to believe that the solute plays a dominating role at such high concentration^50^. In the absence of solute effects, premelted films of thickness $\approx 10\,{\rm nm}$ have been predicted in the particle-front gap by the past studies^15–17^.

Our results depict that an augmentation of solute does not seem to influence the previously predicted value (RW model) of critical radius $R_c$ for droplet engulfment. Similar to the $R_1$ droplets in the presence of $0.01\,$wt.% solute concentration, we report an initial repulsion of the $R_1$ droplets with $1\,$wt.% solute in solution. A few analytical models^20,21,33,34^ and numerical simulations^19,35,49^ studying the object-front interactions in the presence of solute effects have been derived previously. In theory, the magnitude of van der Waals force, responsible for pushing the particle, is regulated by the particle-front gap. The high concentration of segregated solute increases the particle-front gap by constitutional undercooling and hence, significantly reduces the effect of repulsive van der Waals force. This implies that for a given growth rate, smaller objects can be engulfed much more easily in solutions, in contrast to pure melts ($R_c^{~solution} < R_c^{~pure\,melt}$). Thus, the theoretical models predict a critical radius ($R_c$) of magnitude much smaller for binary solutions as compared to the pure melts, which is not what we observe experimentally (i.e. $R_c^{~solution} \approx R_c^{~pure\,melt}$). Therefore, we deduce from our experimental observations that $R_1<R_c<R_2$ is still a valid criterion with $1\,$wt.% solute in solution.

### Interaction of the front with bimodal objects

**Figure 6:**
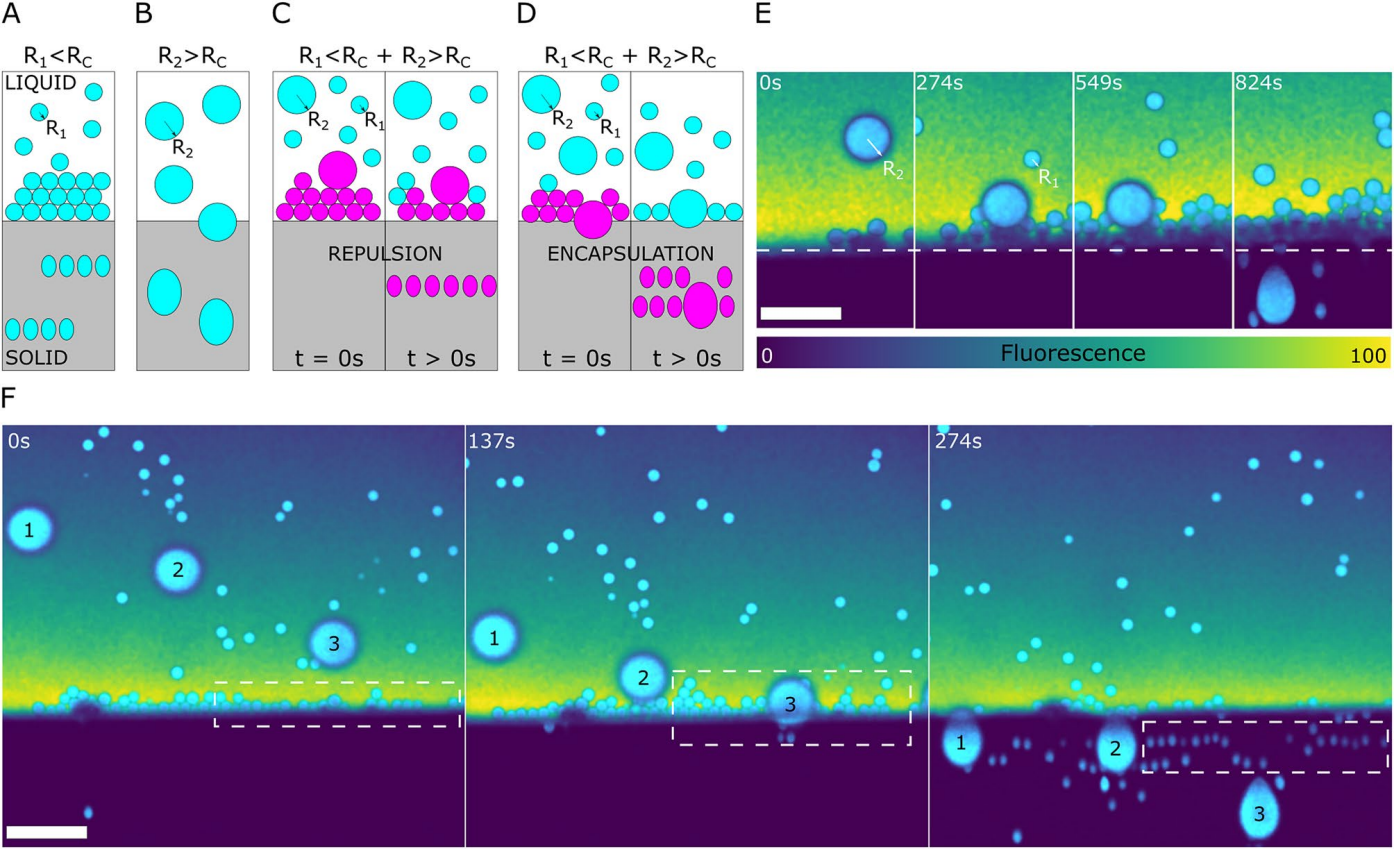
Interaction of bimodal oil-in-water emulsion with an advancing ice-water front with $0.01\,$wt.% bulk solute concentration. (**A**,**B**) Descriptive sketch representing monodisperse emulsion observation as seen previously with (**A**) $8\,\upmu {\rm m}\,(R_1)$ and (**B**) $28\,\upmu {\rm m}\,(R_2)$ droplets (**C**,**D**) Sketch representing the experimental observations with bimodal emulsion, prepared by mixing the $8\,\upmu {\rm m}$ and $28\,\upmu {\rm m}$ droplets (**E**,**F**) Experimental observation of bimodal emulsions depicting (**E**) Repulsion instigated by droplets $R_1<R_c$ forming a barrier layer and pushing droplets with $R_2>R_c$ (**F**) Engulfment instigated of droplets $R_1<R_c$ by droplets with $R_2>R_c$, thereby breaking the compact layer. The droplet 1 is engulfed instantaneously while droplet 2 hits the compact layer and gets repelled before engulfment. Oil is in cyan, water in colormap viridis (fluorescence bar) while ice is in black. Scale bar =$100\,\upmu {\rm m}$. $^{\copyright }$ (2020) S. Tyagi et al. (10.6084/m9.figshare.13176221) CC BY 4.0 license https://creativecommons.org/licenses/by/4.0/.

**Figure 7:**
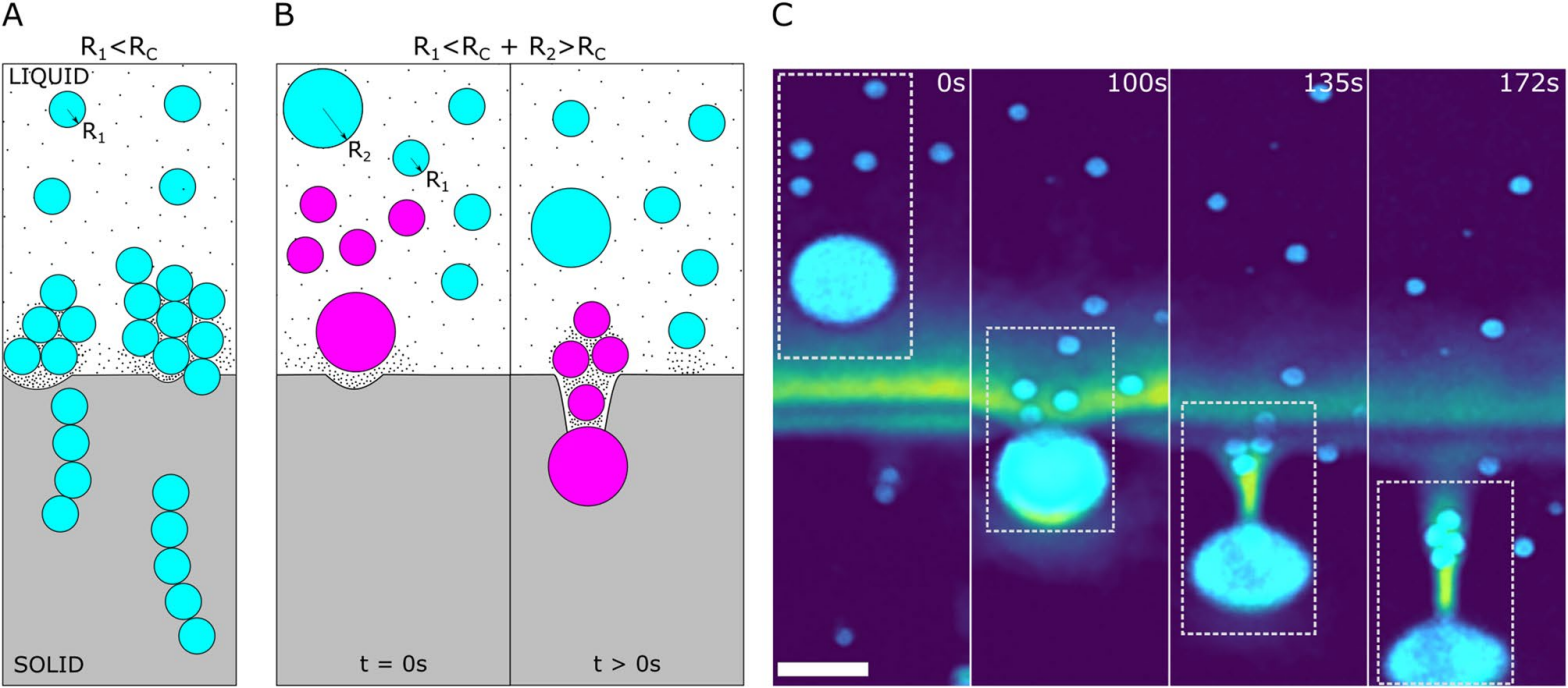
Interaction of bimodal oil-in-water emulsion with an advancing ice-water front with $1\,$wt.% bulk solute concentration. (**A**) Model sketch elucidating the previous experimental observations with $8\,\upmu {\rm m}$ monodisperse droplets ($R_1$) with $1\,$wt.% solute in solution (**B**,**C**) Concave cusping of the solidification front is induced by the rejected solute segregation in the object-front gap and results in the formation of a solute-rich pocket. (**B**) Schematic depicting the formation of a pocket and subsequently induced object clustering with bimodal objects containing $R_1 + R_2$ objects, where $R_1<R_c<R_2$. The small black dots in the panels A and B are a schematic representation of the solute. (**C**) Experimental observation showing engulfment of $8\,\upmu {\rm m}$ oil droplets in pockets initially induced by $28\,\upmu {\rm m}$ droplets. The droplets are far apart in water at $t=0\,{\rm s}$, get clustered at the fermeture of the solute-rich pocket ($t=135\,{\rm s}$), and at $t=172\,{\rm s}$ get further close-packed owing to the lateral growth (along $\vec {y}$) of the ice crystals. Oil is in cyan, water in colormap viridis (fluorescence bar) while ice is in black. Scale bar = $100\,\upmu {\rm m}$. $^{\copyright }$ (2020) S. Tyagi et al. (10.6084/m9.figshare.13176221) CC BY 4.0 license https://creativecommons.org/licenses/by/4.0/.

We have discussed so far the interaction of monodisperse objects, exhibiting distinct radii ($R_1,R_2$), with an approaching solid–liquid front. Yet, in many applications, such as fabrication of metal/ceramic matrix composites, we can have a distribution of object sizes. Thus, it is essential in such scenarios to comprehend and predict, the segregation or sorting of particles, during solidification. In light of these uncertainties, we conducted horizontal unidirectional solidification with the same set of droplets (radii $R_1=8\,\upmu {\rm m}$ and $R_2=28\,\upmu {\rm m}$) composed together, at a solidification velocity $V_{sl}$ of $1\,\upmu {\rm m}\cdot {\rm s}^{-1}$ and a constant linear temperature gradient *G* of $10^4\,{\rm K}\cdot {\rm m}^{-1}$. To understand the novel scenarios observed, we compare the modes of interactions in bimodal emulsions to the monodisperse regimes at their corresponding solute concentrations ($0.01,1\,$wt.%). We also depict the associated descriptive schemes in absence (Fig. 6A–D) and presence (Fig. 7A,B) of dominating solute effects.

In the presence of $0.01\,$wt.% solute (Fig. 6), we notice two peculiar behaviours of the inter-droplet interaction with the solid–liquid front as follows:

First, as shown in Fig. 6C,E the small droplets ($R_1<R_c$) form a compacted layer at the planar front as seen in monodisperse emulsions (Fig. 6A), which further acts as a barrier layer and provokes the repulsion of a larger droplet ($R_2$). The latter is pushed ahead for $t\approx 200\,{\rm s}$ before getting eventually trapped by the growing ice. This suggests that a droplet with a radius greater than the critical value ($R_2>R_c$) can still be repelled if it does not interact with the solidification front immediately. In contrast, isolated monodisperse large droplets get engulfed instantaneously without undergoing repulsion-trapping, as shown schematically in Fig. 6B and experimentally in Fig. 5C.

The second observation (see Fig. 6D,F) depicts the repulsion-trapping transition of the compacted layer ($R_1<R_c$), instigated by the presence of 3 large droplets ($R_2>R_c$), labelled as 1, 2, 3 in Fig. 6F. We also notice the relative repulsion of droplet 2 as compared to droplet 1, similar to Fig. 6C, manifested by the decreasing horizontal distance between $t\approx 0\,{\rm s}$ and $t\approx 274\,{\rm s}$. The droplet 2 encounters a compacted layer in the vicinity of the front, while droplet 1 undergoes instant engulfment owing to its large size ($R_2>R_c$) and an immediate interaction with the solidified crystal. The height (*h*) of the compacted layer is $\approx 90\,\upmu {\rm m}$ at the instance of the repulsion-trapping transition in the two observations stated above (Fig. 6E,F). Interestingly, this corresponds closely to the height of $\approx 80\,\upmu {\rm m}$, observed in Fig. 5A with monodisperse small droplets. The trapping of droplets, for a sufficiently large thickness of the compacted layer, prevents their accumulation and thereby ensures a constant steady-state layer thickness^32^. This clearly demonstrates the importance of multiple-particle interactions with a solid–liquid front on the development of a solidified microstructure.

We now investigate the dynamics of bimodal droplet size distribution with $1\,$wt.% solute in solution. The large droplet ($R_2=28\,\upmu {\rm m}$) as shown in Fig. 7B,C provokes a concave curvature ($t=0\,{\rm s}$), thereby diminishing the crystal growth rate behind it. The pocket created in the ice proceeds with the engulfment of the large droplet surrounded by a relatively high concentration of solute ($t=100\,{\rm s}$). The temporary opening, at the solid–liquid front, entices a cluster of smaller droplets ($R_1=8\,\upmu {\rm m}$) behind the large droplet ($t=135\,{\rm s}$). The droplet cluster, initially isolated, is trapped at the fermeture of the pocket and gets textured perpendicular to the front ($\vec {x}$). This orientation is reminiscent of a solidifying monodisperse emulsion depicted schematically in Fig. 7A. The lateral growth of the ice crystals, transverse to the temperature gradient, pushes the droplets together and results in a close-packed microstructure. Thus, a small increase in the amount of solute added has again a drastic impact on the solidification microstructure.

### Interaction of the front with polydisperse objects

**Figure 8:**
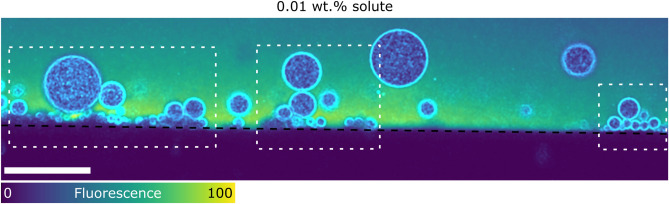
Interaction of polydisperse oil-in-water emulsion with an advancing ice-water front at $0.01\,$wt.% bulk solute concentration. The droplets with radii smaller than the critical radius ($R_x<R_c$) get pushed by the steady-state planar front and lead to the formation of clusters by blocking the arriving droplets ($R_x<R_c<R_y$). Oil is in cyan, water in colormap viridis (fluorescence bar) while ice is in black. Scale bar = $100\,\upmu {\rm m}$. $^{\copyright }$ (2020) S. Tyagi et al. (10.6084/m9.figshare.13176221) CC BY 4.0 license https://creativecommons.org/licenses/by/4.0/.

**Figure 9:**
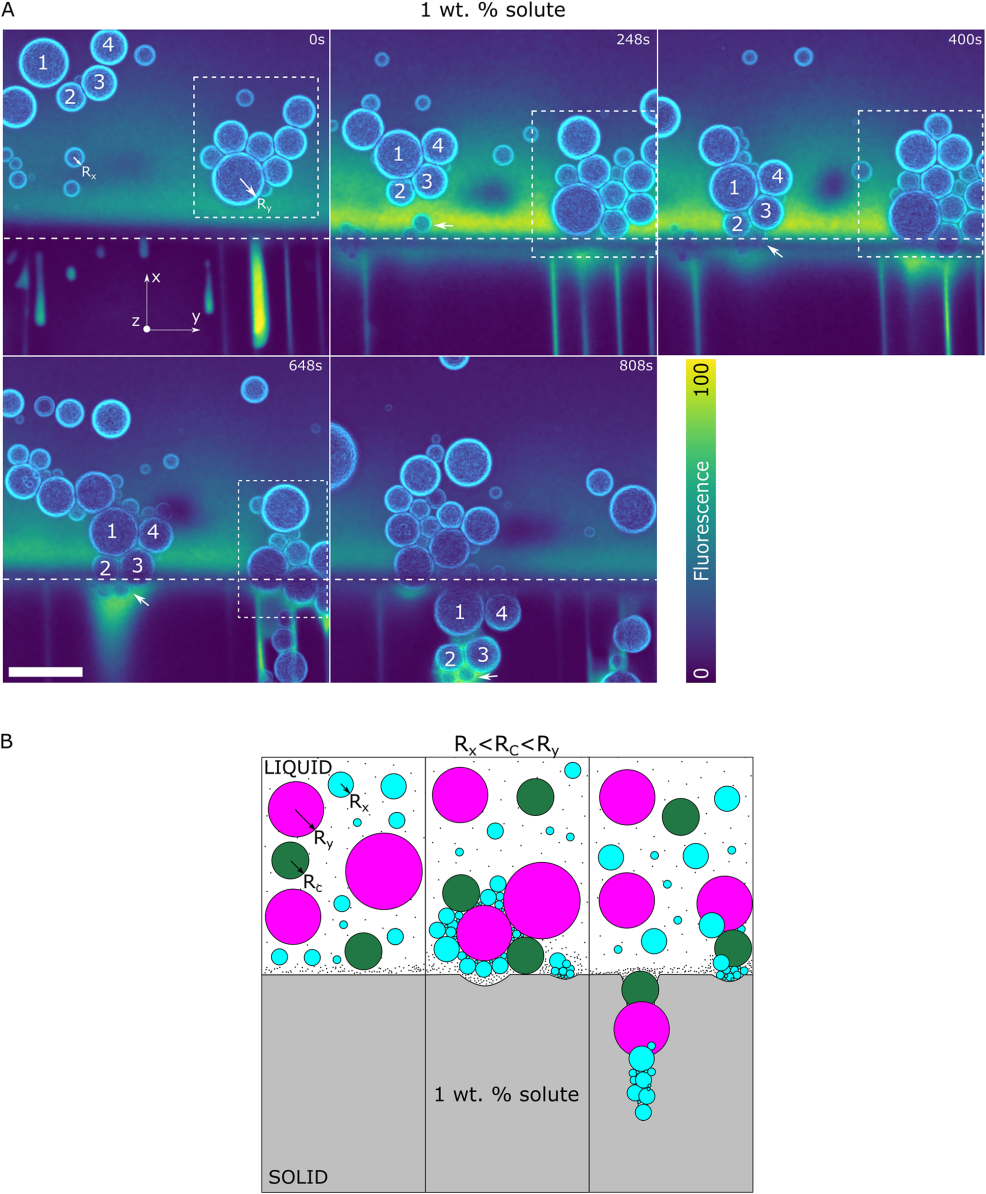
Interaction of polydisperse oil-in-water emulsion with an advancing ice-water front at $1\,$wt.% bulk solute concentration. (**A**) Droplets 1,2,3,4 form a cluster at the ice-water front, while the droplets in the insets form a cluster ahead of the front. The agglomerates depict a pushing-engulfment transition with local destabilisation of the ice-water front (**B**) Schematic depicting the experimental observations in (**A**), where small droplets ($R_x<R_c$) form a barrier and subsequently form close-packed agglomerates with a combination of droplet sizes ($R_x<R_c<R_y$) before getting captured in the solid. Oil is in cyan, water in colormap viridis (fluorescence bar) while ice is in black. Scale bar = $100\,\upmu {\rm m}$. $^{\copyright }$ (2020) Tyagi et al. (10.6084/m9.figshare.13176221) CC BY 4.0 license https://creativecommons.org/licenses/by/4.0/.

In the last section, we look at the behaviour of polydisperse oil-in-water emulsions. The low interfacial tension of the oil (propyl benzoate) with water enables us to obtain relatively small polydisperse droplets, varying in size from $5< R < 30\,\upmu {\rm m}$. We utilize in situ cryo-confocal microscopy to acquire 2D and 3D evolution of the microstructure at $V_{sl}=1\,\upmu {\rm m}\cdot {\rm s}^{-1}$ and $G=10^4\,{\rm K}\cdot {\rm m}^{-1}$. Previously, we have reported on the monodisperse and bimodal size particle interactions between the growing solid and the droplets, which is considerably different from the nature of interactions between the front and an isolated object. To attain a better understanding of complex (more realistic) systems, we will now investigate the confrontation of polydisperse droplets with an advancing solidification front. The emulsions are prepared with $0.01\,$wt.% and $1\,$wt.% solute in solution.

When an object interacts with a solid–liquid front it can be engulfed immediately, pushed irreversibly, or repelled for a certain distance and then engulfed^15^. These behaviours are encountered owing to a critical velocity ($V_c$), inversely proportional to the particle radius ($V_c\propto R^{-\alpha }$), above which an object is engulfed ($V_{sl}>V_c$), while below it the object is pushed by the front ($V_{sl}<V_c$). Conversely, for a given growth rate $V_{sl}$, there should exist a critical radius $R_c$, where the objects smaller than the critical size should be pushed and vice-versa. Thus, in our experiments with different object sizes one would expect a range of critical velocities or a range of critical radii at the imposed growth rate. Ideally, we expect the engulfment of objects with $R_y>R_c$ and rejection of objects with $R_x<R_c$, where the critical radius is $9\,\upmu {\rm m}$ for our experimental parameters. However, we observe the repulsion of miscellaneous droplets irrespective of their radii ($R_x<R_c<R_y$), as seen in Fig. 8, in the absence of long-range solute effects. The droplet clusters form at the front with the primary layer occupied by the small droplets ($R_x<R_c$), which further creates a barrier and facilitate the repulsion of relatively larger droplets ($R_y>R_c$).

For planar front solidification in the presence of solute, the previous models^19,21,33,34^ deduce a critical engulfment velocity which in most cases is an order below the critical velocity for isolated objects in pure materials. The models suggest either an absence of pushing-engulfment transition, or a destabilisation of the steady-state planar front prior to engulfment. In both scenarios, rapid engulfment of the object is suggested as the most favorable outcome, facilitated by an increase in the viscous drag forces. However, the presence of multiple-particle interactions is not incorporated in these models. The experimental evidence are difficult to correlate with owing to varying process parameters and often suggest contradictory results^4,23–25^. Körber et al.^4^ have reported no impact of solute on critical velocity in water-NaMnO$_4$ solution, in contrast to the theoretically expected decrease in $V_c$ (from pure melts) owing to the impurity effects. On the contrary, Sekhar & Trivedi^23^ have demonstrated strong impacts of impurity leading to particle trapping in the directional solidification of succinonitrile-acetone system.

In our experiments, we report a pushing-engulfment or repulsion-trapping transition in the presence of dominating solute effects. From Fig. 9A, we observe the presence of droplet clusters generated both at (inset 1,2,3,4 in Fig. 9A) and ahead of the front (inset box in Fig. 9A). The 3D time-lapse evolution depicts the small droplet (inset arrow Fig. 9A) being pushed by the front for $\approx 400\,{\rm s}$. Consequently, the droplets (inset 1,2,3,4 in Fig. 9A) are blocked and form a close-packed cluster, eventually engulfed at $t= 808\,{\rm s}$. We illustrate this scenario in Fig. 9B with a model schematic. The morphology of the front does not undergo a transformation in the presence of droplets, as suggested previously^23,51,52^, and remains as depicted in the absence of objects (Fig. 4). The formation of the cluster ahead of the front is still not clear but we believe the solute phoretic phenomenon such as diffusiophoresis can be at the genesis of such long-range displacement^37^. Although, we require more experiments to conclude effectively the origin of such close-packed clusters.

## Conclusions

The size, presence of multiple objects, complex front morphology, and high concentration of solute significantly alter the microstructure developed during directional solidification. We performed solidification experiments to study the droplet rearrangement of monodisperse, bimodal, and polydisperse distributions in the solidified matrix. We have tried to highlight the correlations and disparities of multiple-particle regime with previous isolated single object models^16,19^ as well as recent multi-particle models^32^, computed both in the absence and presence of solute effects. We have successfully demonstrated in our experiments the distinct behaviour when the size of droplets adheres to the criterion of $R_1<R_c<R_2$. Furthermore, we have observed no change in critical radius ($R_c$) in the presence of solute effects contrary to the predictions of the theoretical models. The critical radius ($R_c$) or critical velocity ($V_c$) is modified by the presence of inter-droplet interactions. We depict that the presence of multiple objects can lead to the formation of a segregated microstructure, while the initial suspension is homogeneous. We illustrate for the first time a pushing-engulfment or repulsion-trapping transition in the presence of overriding solute effects. We report no change in the morphology of the solidification front owing to the presence of objects in the melt. We suggest that the volume fraction of objects in the melt is an important criterion to be considered for predicting the object distribution in the solidified microstructure. The solidification of controlled oil-in-water emulsions can help us visualize and model a variety of microstructures by utilizing different colonies of droplets in the presence or absence of solute effects.

## Data Availability

The datasets generated during and/or analysed during the current study are available from the corresponding author on reasonable request.
